# Human Adipose Tissue-Derived Stromal/Stem Cells Promote Migration and Early Metastasis of Triple Negative Breast Cancer Xenografts

**DOI:** 10.1371/journal.pone.0089595

**Published:** 2014-02-28

**Authors:** Brian G. Rowan, Jeffrey M. Gimble, Mei Sheng, Muralidharan Anbalagan, Ryan K. Jones, Trivia P. Frazier, Majdouline Asher, Eduardo A. Lacayo, Paul L. Friedlander, Robert Kutner, Ernest S. Chiu

**Affiliations:** 1 Department of Structural and Cellular Biology, Tulane University School of Medicine, New Orleans, Louisiana, United States of America; 2 Stem Cell Biology Laboratory, Pennington Biomedical Research Center, Louisiana State University, Baton Rouge, Louisiana, United States of America; 3 Department of Otolaryngology, Tulane University School of Medicine, New Orleans, Louisiana, United States of America; 4 Gene Therapy Program, Louisiana State University Health Sciences Center, New Orleans, Louisiana, United States of America; 5 Department of Plastic Surgery, New York University Langone Medical Center, New York, New York, United States of America; National Cancer Institute, United States of America

## Abstract

**Background:**

Fat grafting is used to restore breast defects after surgical resection of breast tumors. Supplementing fat grafts with adipose tissue-derived stromal/stem cells (ASCs) is proposed to improve the regenerative/restorative ability of the graft and retention. However, long term safety for ASC grafting in proximity of residual breast cancer cells is unknown. The objective of this study was to determine the impact of human ASCs derived from abdominal lipoaspirates of three donors, on a human breast cancer model that exhibits early metastasis.

**Methodology/Principal Findings:**

Human MDA-MB-231 breast cancer cells represents “triple negative” breast cancer that exhibits early micrometastasis to multiple mouse organs [1]. Human ASCs were derived from abdominal adipose tissue from three healthy female donors. Indirect co-culture of MDA-MB-231 cells with ASCs, as well as direct co-culture demonstrated that ASCs had no effect on MDA-MB-231 growth. Indirect co-culture, and ASC conditioned medium (CM) stimulated migration of MDA-MB-231 cells. ASC/RFP cells from two donors co-injected with MDA-MB-231/GFP cells exhibited a donor effect for stimulation of primary tumor xenografts. Both ASC donors stimulated metastasis. ASC/RFP cells were viable, and integrated with MDA-MB-231/GFP cells in the tumor. Tumors from the co-injection group of one ASC donor exhibited elevated vimentin, matrix metalloproteinase-9 (MMP-9), IL-8, VEGF and microvessel density. The co-injection group exhibited visible metastases to the lung/liver and enlarged spleen not evident in mice injected with MDA-MB-231/GFP alone. Quantitation of the total area of GFP fluorescence and human chromosome 17 DNA in mouse organs, H&E stained paraffin sections and fluorescent microscopy confirmed multi-focal metastases to lung/liver/spleen in the co-injection group without evidence of ASC/RFP cells.

**Conclusions:**

Human ASCs derived from abdominal lipoaspirates of two donors stimulated metastasis of MDA-MB-231 breast tumor xenografts to multiple mouse organs. MDA-MB-231 tumors co-injected with ASCs from one donor exhibited partial EMT, expression of MMP-9, and increased angiogenesis.

## Introduction

Approximately 120,000 patients diagnosed with breast cancer undergo partial mastectomy and radiation therapy each year. While this treatment plan is suggested to be equally effective in patient survival compared to complete mastectomy, it typically results in breast distortion and asymmetry due to avascular fibrosis and breast tissue atrophy. Subsequent radiation treatment may result in fibrosis, chronic ischemia and hypoxia leading to poor wound healing and major discomfort and loss of motion. Fat grafting provides volume replacement, but may also improve the quality of surrounding damaged skin and subcutaneous tissue.

Grafted adipocytes serve as ideal filler. Adipocytes are autologous, available in sufficient quantities in most patients, and are potentially permanent. It is for these reasons that fat grafting has gained popularity for soft tissue repair [2]–[14]. In any given fat harvest site (abdomen, flank, buttock, etc.), there is an ample supply of adipocytes and a small percentage of adipose tissue-derived stromal/stem cells (ASCs). ASCs have potent angiogenic and regenerative properties capable of treating numerous medical and surgical problems [13], [15], [16]. It has been proposed that supplementing fat grafts with ASCs will improve graft retention and the regenerative/restorative ability of the graft. A number of case reports and small studies suggested that fat grafts supplemented with ASCs for breast reconstruction did not impact breast tumor recurrence rates after short term follow up [17]–[20], [20]–[24]. A recent prospective multi-center clinical trial of 67 breast cancer patients treated with ASC-supplemented fat grafts to repair surgical defects found no local tumor recurrence 12 months after treatment [25]. Despite the potential of ASC supplementation fat grafts, long term follow-up studies to determine safety have not been performed and controversy remains regarding ASC supplementation of fat grafts [26]–[28].

Laboratory studies to measure effects of ASCs or related bone marrow-derived mesenchymal stem cells (MSCs) on primary breast cancer growth or metastasis has yielded mixed results that is likely due to different sources of ASCs used (mouse or human, abdominal/visceral, bone or reduction mammoplasty-derived), donor effects, and the use of different tumor models. Studies using established breast cancer cell lines or pleural effusions from breast cancer patients demonstrated that either co-culture with ASCs or conditioned medium (CM) from ASCs promoted growth, and/or epithelial to mesenchymal transition (EMT) and invasion of breast cancer cells *in vitro* [29]–[33], [33]–[35]. In addition, co-injection of ASCs with breast cancer cells promoted primary tumor xenograft growth in mice [29], [31], [36] and altered the makeup and stiffness of the tumor stroma [37]–[39]. Cancer initiating cells derived from the murine 4T1 mammary cancer cell line that were co-injected with murine ASCs increased primary tumor volume 2.5 fold with an increase in lung metastasis that was secondary to the increased tumor volume [31]. Conversely another study using human ASCs isolated from reduction mammoplasty tissue found that ASCs inhibited primary breast cancer xenograft growth and metastasis [40]. Studies of MSCs derived from bone marrow have also shown mixed results on breast cancer growth and metastasis *in vitro* and *in vivo* [41]–[45]. MSCs may have more significant impact on promoting tumor metastasis than effects on primary tumor growth [42], [46] by inducing an EMT in cancer cells. Taken together, these studies suggested that MSCs and ASCs may promote the initial stages of breast cancer metastasis by promoting a metastatic phenotype in the breast cancer cells, and possibly by altering or breaking down the tumor extracellular matrix.

The objective of the present study was to determine the impact of ASCs likely to be employed for grafting procedures on breast cancer growth and metastatic incidence, rate and organ specificity. The study used well-characterized human ASCs derived from subcutaneous abdominal adipose tissue from three healthy female donors. To study the effect of ASCs on early metastasis, this study used the human MDA-MB-231 breast cancer model which does not express estrogen receptor (ER), progesterone receptor (PR) or Her2 [47] and represents a model of “triple negative” breast cancer in patients, a more aggressive and metastatic breast cancer subtype. We developed a xenograft procedure for MDA-MB-231 tumors that resulted in early metastasis to multiple mouse organs within 30–40 days [1].

## Materials and Methods

### Ethics Statement

Subcutaneous abdominal adipose tissue from healthy female patients was obtained during elective surgery with the patient's informed, written consent under a protocol approved by the Institutional Review Board of the Pennington Biomedical Research Center Institution. Consent was obtained by the plastic surgeons performing the surgery and tissues were provided to the investigators with all identifying information removed. All mouse experiments were performed in accordance with approved IACUC protocol (#2941R2) from Tulane University.

### Materials

All chemicals were purchased from Sigma-Aldrich (St. Louis, MO) or Fisher Scientific (Norcross, GA) unless otherwise specified.

### Isolation, collection, and culture of human ASCs

ASCs were isolated as described [15], [48] from the abdominal lipoaspirates from three female donors with mean age 39.7±9.1 and mean BMI 22.3±2.9. The mean percentage of ASCs that were positive for individual surface markers were as follows: CD29, 99.3±0.3; CD105, 98.4±0.9; CD45, 12.2±2.9; CD34, 95.3±1.0; CD44, 12.6±2.6; CD73, 91.4±2.6; CD90, 94.8±1.6. Passage 0 ASC were expanded in cell factories in ASC growth medium [DMEM/F-12 Ham's, 10% FBS (Hyclone, Logan, UT, http://www.hyclone.com), 1% Penicillin-Streptomycin/0.25 g fungizone] and cryopreserved in cryopreservation medium [10% dimethylsulfoxide, 10% Dulbecco's modified Eagle's medium (DMEM)/F-12 Ham's, 80% calf serum], frozen at 80°C in an ethanol-jacketed closed container, and subsequently stored in liquid nitrogen prior to thawing for individual assays. All experiments were performed using passage 1 (P1) ASCs that were reconstituted from cryopreserved P0 ASCs and cultured in ASC growth medium on polystyrene tissue culture dishes as described [15], [48]. To collect conditioned medium from ASC in growth medium (growth conditioned medium (GCM)), ASCs were cultured until 40% confluency, the medium was replaced with the same medium containing only 2% FBS, and the cells were cultured for an additional three days before collection of the GCM that was stored at 4°C until use.

### Adipogenic differentiation of ASCs

Adipogenic differentiation of ASCs was performed as previously described [49]. Briefly, ASCs were cultured in ASC growth medium until the culture reached 90–95% confluency. ASCs were then trypsinized and plated in 24-well plates in ASC growth medium at 30,000 cells/cm^2^ for 24 hrs. to allow attachment. On day 1 (24 hours after plating), the medium was removed and cells were incubated for three days in ASC adipogenic differentiation medium [Dulbecco's modified Eagle's-Ham's F-12 medium supplemented with 3% or 10% FBS, 15 mM HEPES (pH 7.4), biotin (33 µM), pantothenate (17 µM, Sigma), human recombinant insulin (100 nM, Boehringer Mannheim), dexamethasone (1 µM), 1-methyl-3-isobutylxanthine (IBMX; 0.25 mM), and rosiglitazone (1 µM)]. For the remaining 9 days of the adipocyte differentiation maintenance period, the medium was removed every 3 days and replaced with the same medium that did not contain IBMX and rosiglitazone (maintenance medium). Adipocyte differentiated conditioned medium (ADCM) was collected on day 6 after the switch to ASC adipogenic differentiation medium and stored at 4°C until use.

### Preparation of ASC/RFP cells

P0 ASCs (isolated from female donor age 44, BMI 24.98) were plated at a density of 5×10^4^ cells/well in a 6-well plates in ASC growth medium and incubated at 37°C in the presence of 5% CO_2_ for 24 hours in a biological safety cabinet. The medium was replaced with 1.5 mL fresh medium containing 8 µg/ml polybrene to increase lentivirus transduction efficiency. ASCs were transduced by addition of 5 µl RFP-lentiviral vector stock, NL-Turbo-RFP (MOI in the range of >10^7^ TU/mL) [50]. After 24 hour incubation, medium containing lentiviral particles was removed and 2 ml fresh medium was added to the wells. ASC/RFP cells were passaged over a period of 2–3 weeks until >90% transduction was visually observed by fluorescent microscopy for RFP using a Nikon microscope with the filter for red fluorescence (TRITC).

### Culture of MDA-MB-231 and MDA-MB-231/GFP cells

MDA-MB-231 cells were purchased from American Type Culture Collection (ATCC, Manassas, VA) and MDA-MB-231/GFP cells were purchased from Cell Biolabs, Inc. (San Diego, CA). Cells were cultured in DMEM supplemented with 10% FBS and 1% Penicillin-Streptomycin, and incubated at 37°C in the presence of humidified 5% CO_2_ incubator.

### Indirect co-culture of ASCs with MDA-MB-231 cells

The effect of ASC on MDA-MB-231 growth and migration was assessed using BD Invasion Chambers and Control Inserts (BD Biosciences, San Jose, CA) according to manufacturer instructions. To measure MDA-MB-231 growth, 2.5×10^4^ MDA-MB-231 cells were plated in the bottom chamber and 2.5×10^4^ cells ASCs were plated in the control inserts in DMEM supplemented with 2% FBS and 1% Penicillin-Streptomycin. The chambers were incubated for 1–4 days in a 37°C incubator with humidified 5% CO_2_. MDA-MB-231 growth in the wells was assessed using reduction of 3-(4-5-Dimethylthiazol-2-yl)-2, 5-diphenyltetrazolium bromide (MTT, Invitrogen). To assess MDA-MB-231 migration, MDA-MB-231 cells were plated in the insert and the ASCs were plated in the bottom chamber and the chambers were incubated for 1–4 days. Membranes were dissected out of chambers and the membranes stained with crystal violet followed by quantification of color development. Analysis of the plates and the inserts were performed at 24 hrs., 48 hrs., 72 hrs., and 96 hrs. following plating. At least three independent sets of experiments were performed using three ASC donors.

### Direct co-culture of ASCs with MDA-MB-231/GFP cells

2.5×10^4^ASCs were cultured in ASC growth medium in triplicate in 6 well plates for 24 hrs. prior to addition of 2.5×10^4^ MDA-MB-231/GFP breast cancer cells to the same wells. Bright field and fluorescent microscopy photographs were taken with a Nikon AZ100 fluorescent microscope and photomicrographed with a Nikon DS-Qi1Mc camera on days 1–4 after addition of the MDA-MB-231/GFP cells. The average number of GFP^+^ cells counted in four separate fields was recorded using the threshold adjustment and particle analysis tools on ImageJ software (NIH, Bethesda, MD). At least three independent sets of experiments were performed using three ASC donors.

### Wound healing/scratch assay to measure effect of ASC conditioned medium on MDA-MB-231 migration

MDA-MB-231 cells were cultured to 80% confluency on 12-well plates in DMEM supplemented with 2% FBS and 1% Penicillin-Streptomycin. After 24 h, the medium was replaced with fresh medium containing 0%, 20% or 50% GCM or ADCM from ASCs. A single strip of cells was scraped off the surface of the plate with a 200 µl disposable plastic pipette tip and the cells were cultured for an additional 6 hours at 37°C. Wound closure was viewed under a microscope and photographed (original magnification, ×40). The average extent of wound closure was evaluated by measuring the width of the wound by Image J software. Percent gap closure was calculated as
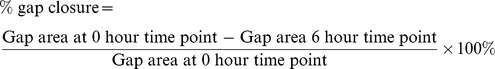



### Animals

Female NUDE mice (BALB/c) aged between 4–5 weeks obtained from Charles River (Indianapolis, IN) were housed in sterile cages and maintained in pathogen-free aseptic rooms with 12 h/12 h light/dark schedule. Mice were fed with autoclaved food pellets and water *ad libitum*.

### Tumor xenograft studies

Xenograft procedures were performed as previously described by our laboratory [1], [51]–[53]. Briefly, exponentially growing MDA-MB-231/GFP cells and ASC/RFP cells were harvested. Animals were divided into 3 groups (n = 5 mice/group, 10 tumors/group) by injecting either 3×10^6^ MDA-MB-231/GFP cells, 3×10^6^ ASC/RFP cells or MDA-MB-231/GFP+ASC/RFP in 150 µl of PBS-Matrigel mixture (50 µl cell suspension in PBS was mixed with 100 µl of Matrigel) orthotopically and bilaterally into the inguinal mammary fat pads of female NUDE mice. In all experiments, tumor caliper measurements were taken twice/week and tumor volume was calculated by the formula: 0.523×LM^2^ where L is (large diameter) and M is small diameter as described [51], [52]. 40 days post injection, mice were euthanized by exposure to CO_2_ and tumors and mouse organs were removed for further evaluation.

### Fluorescence microscopy and hematoxylin & eosin (H&E) staining of tumors and mouse tissues

At the end of the experiments, animals were sacrificed and tumors and mouse organs removed. Immediately after removal, fresh tumors were placed on a Nikon AZ100 fluorescent microscope and photomicrographed with a Nikon DS-Qi1Mc camera using NIS-Elements software. Subsequently, the tumors were either stored in 10% neutral buffered formalin for paraffin embedding/sectioning and H&E staining, snap frozen for measurement of chromosome-17 by real-time RT-PCR, or embedded for frozen sectioning and fluorescent microscopy as described in our previous studies [1], [51]–[53]. Paraffin embedded tumor and mouse tissues were sectioned (5 µm) and stained with H&E. For immunofluorescence, pieces of tumor and mouse tissues were embedded in O.C.T. (Optimal Cutting Temperature) compound and 10 µm frozen sections were incubated with DAPI for 5 minutes to stain nuclei blue and subsequently prepared for fluorescent microscopy of GFP and RFP and photomicrographed using a Nikon DS-Qi1Mc camera using NIS-Elements software.

### Quantification of micrometastases

To detect micrometastasis, DNA from mouse organs was extracted using the QIAamp DNA mini kit (Qiagen) and quantified using a NanoDrop Spectrophotometer (ThermoScientific). Human DNA in mouse organs was detected by quantitative real time RT-PCR using primer and probes directed towards a human-specific α-satellite DNA sequence of the centromere region of human chromosome 17. To detect micrometastasis, DNA from mouse organs of both the MDA-MB-231/GFP and MDA-MB-231/GFP+ASC/GFP groups were extracted using the QIAamp DNA mini kit (Qiagen) and quantified using a NanoDrop Spectrophotometer (ThermoScientific). Human DNA in mouse organs was detected by quantitative real time RT-PCR using primer and probes directed towards a human-specific α-satellite DNA sequence of the centromere region of human chromosome 17 [54] as we have previously described [1]. Genomic DNA that was isolated from MDA-MB-231 human breast cancer xenografts and organs of nude mice with no human cells injected was used as positive control and negative controls, respectively. Quantitative real-time PCR was performed in a volume of 25 µl that contained 12.5 µl FastStart Taqman Probe Master for probes (Roche), 200 nmol/L each of the forward and reverse primers, 100 nmol/L TaqMan probe, and 250 ng target DNA template. Reactions were incubated at 50°C for 2 minutes and at 95°C for 10 minutes, followed by 40 cycles at 95°C for 15 seconds, and 60°C for 1 minute using a Bio-Rad iQ5 Multicolor Real-Time PCR Detection System (Bio-Rad). Real time RT-PCR for human/mouse GAPDH were performed in a volume of 25 µl that contained 12.5 µl iQ SYBR Green Supermix (Bio-Rad), 900 nmol/L each of the forward and reverse primers and 250 ng target DNA template. All real-time PCR assays were performed in triplicate.

Human Cr17_1a forward primer: 5′-GGG ATA ATT TCA GCT GAC TAA ACA G-3′

Human Cr17_4b reverse primer: 5′-AAA CGT CCA CTT GCA GAT TCT AG-3′

TMsat_probe : 6FAM-CAC GTT TGA AAC ACT CTT

XT TTG CAG GATC p (X = Tamra)

Mouse/human GAPDH forward primer: 5′- CAG CGA CAC CCA CTC CTC CAC CTT -3′

Mouse/human GAPDH reverse primer: 5′- CAT GAG GTC CAC CAC CCT GTT GCT -3′

The CT value obtained for human chromosome 17 was normalized using primers and probe that detected both mouse and human GAPDH as a measure of total DNA for the samples. deltaCT = CT value of human chromosome-17 minus CT value of mouse/human GAPDH. For incidence of metastasis, a deltaCT value below 25 was scored positive for metastasis to the mouse organ/tissue. For comparison of metastasis to different organs/tissue between groups, the data was presented as, fold change = 2^−(delta-deltaCT)^ where MDA-MB-231/GFP alone was set as 1, and delta-deltaCT = deltaCT of MDA-MB-231/GFP+ASC/RFP minus deltaCT of MDA-MB-231/GFP alone

### Detection of metastasis in whole organs by fluorescence quantitation

Metastases in fresh, whole organs were quantitated as described [55] by detection of green fluorescence using a Nikon AZ100 fluorescence microscope with a Nikon AZ100 Plan Fluor 5× objective. Fluorescent pseudocolored images representing light emitted from metastatic sites were captured using Nikon DS-Qi1Mc digital camera. Image J software was employed to quantitate the area of fluorescent signal on the image. The following sequence of events for the acquired jpeg images were executed: 1) Open image. 2) Type Convert to 8 bit image. 3) Edit Invert image. 4) Analyse Set scale unit length µM using known distance. 5) Set threshold. 6) Analyze Set measurements check the area and select Analyze and measure area. 7. Save information (total area, and area fraction) (http://rsbweb.nih.gov/ij/docs/pdfs/examples.pdf). Statistical analysis using Student's t-test was performed.

### Immunohistochemistry (IHC)

IHC staining was performed on 10% neutral buffered formalin fixed paraffin-embedded tumor samples as described previous [1], [51], [56]. Briefly, sections mounted on slides were deparaffinized in xylene, dehydrated in ethanol, rinsed in water and antigen retrieval was carried out with 0.01 M citrate buffer (pH 6.0) for 20 min in a steamer and then incubated with 3% hydrogen peroxide for 5 min. After washing with PBS, sections were blocked by incubation in 10% normal goat serum for 30 min, followed by overnight incubation with primary antibody. The source of the primary antibody and the dilutions used for IHC are as follows, vimentin (1∶100; Vector labs, Burlingame, CA), E-cadherin (1∶400; Cell signaling Technology Inc., Danvers, MA), β-catenin (1∶800; Cell signaling Technology Inc., Danvers, MA), CD-31 (1∶50; Abcam, Cambridge, MA), MMP-2, (1∶250; Abcam, Cambridge, MA), MMP-9 (prediluted ready-to-use; Neomarkers, Fremont, CA), IL-8 (1∶500; Invitrogen, Camarillo, CA), and VEGF (1∶100; Biocare Medical, Concord, CA). After overnight incubation with primary antibody, slides were washed with PBS followed by 30 minutes incubation with biotinylated secondary antibody (Vector labs), rinsed in PBS and incubated with ABC reagent (Vector labs) for 30 min. The stain was visualized by incubation in 3, 3-diaminobenzidine (DAB) and counterstained with Harris hematoxylin. Internal negative control samples incubated with either non-specific rabbit IgG, or 10% goat serum instead of the primary antibody showed no specific staining. Slides were dehydrated and mounted with Permount (Fisher). Slides were visualized using a Nikon OPTIPHOT microscope and randomly selected bright field microscope images (magnification, ×200) were captured by Nikon Digital Sight High-Definition color camera (DS-Fi1) using NIS-Elements BR software. IHC staining intensity was scored using the histoscore method developed by Allred et al., 1993 [57] and as we have previously described [1], [51], [56].

### Statistical Analysis

Statistical analysis of the data was performed using Graphpad Prism v5.0 software. Data were expressed as mean +/−SD. P<0.05 was considered significant. The mean and S.D. were calculated using Microsoft Excel or GraphPad Prism 5 software (La Jolla, CA). Statistical significance was determined by two-sample student t-tests (P<0.05) (two-tailed) and one-way ANOVA followed by Newman-Keuls multiple comparison test.

## Results

### ASC effect on growth and migration of breast cancer cells *in vitro*

**Figure 1:**
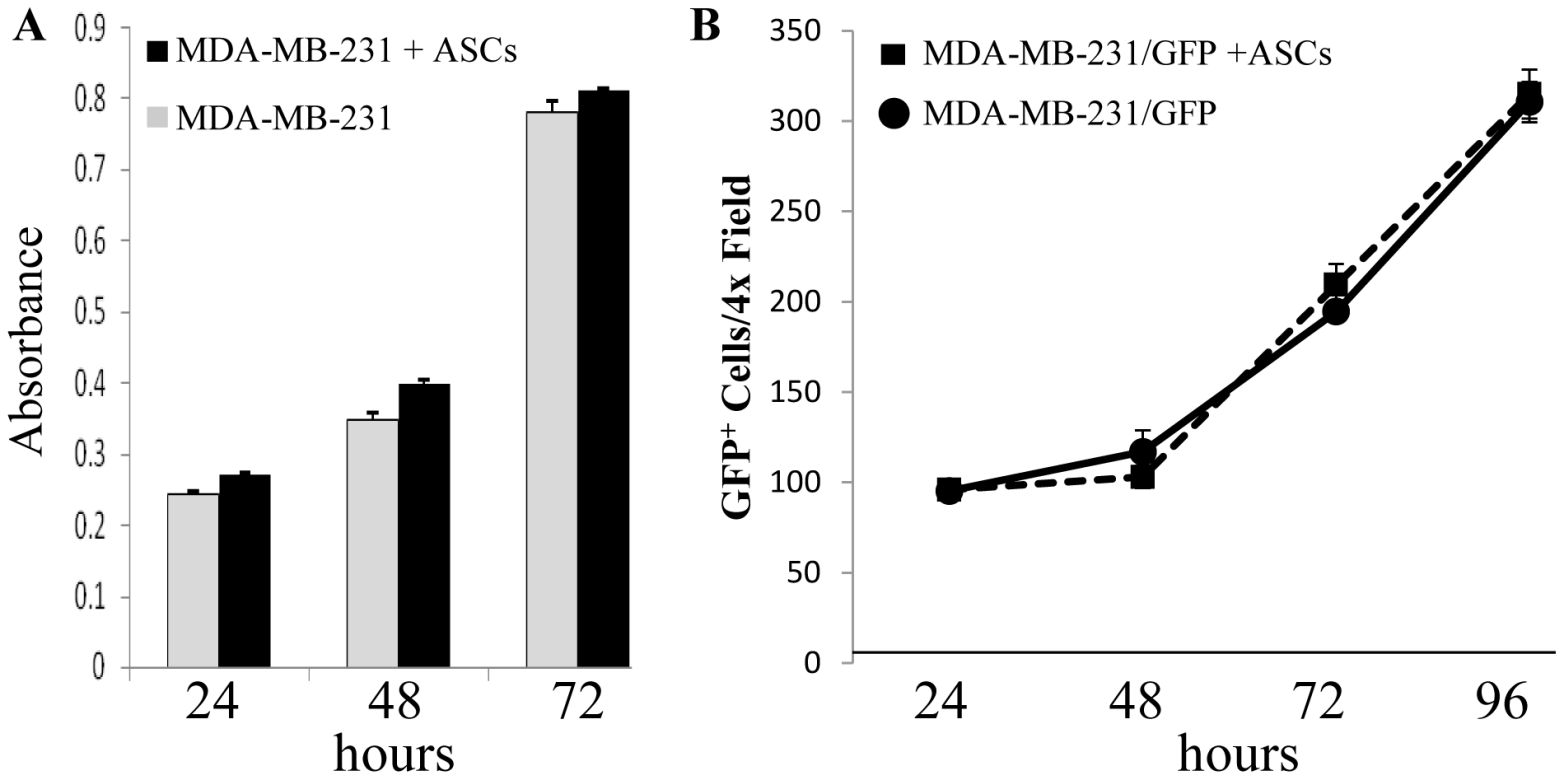
The effect of ASCs on the growth of MDA-MB 231 cells. A. MDA-MB-231 were cultured in the bottom well of a Boyden Chamber and ASCs were cultured in the insert. Growth of MDA-MB-231 cells was assessed using the MTT assay. **B.** 2.5×10^4^ASCs were cultured in 6 well plates for 24 hrs. prior to addition of MDA-MB-231-GFP breast cancer cells at a 1∶1 ratio. Bright field and fluorescent microscopy photographs were taken on days 1–4 after addition of the MDA-MB-231 cells. Data are representative of experiments using three different ASC donors.

**Figure 2:**
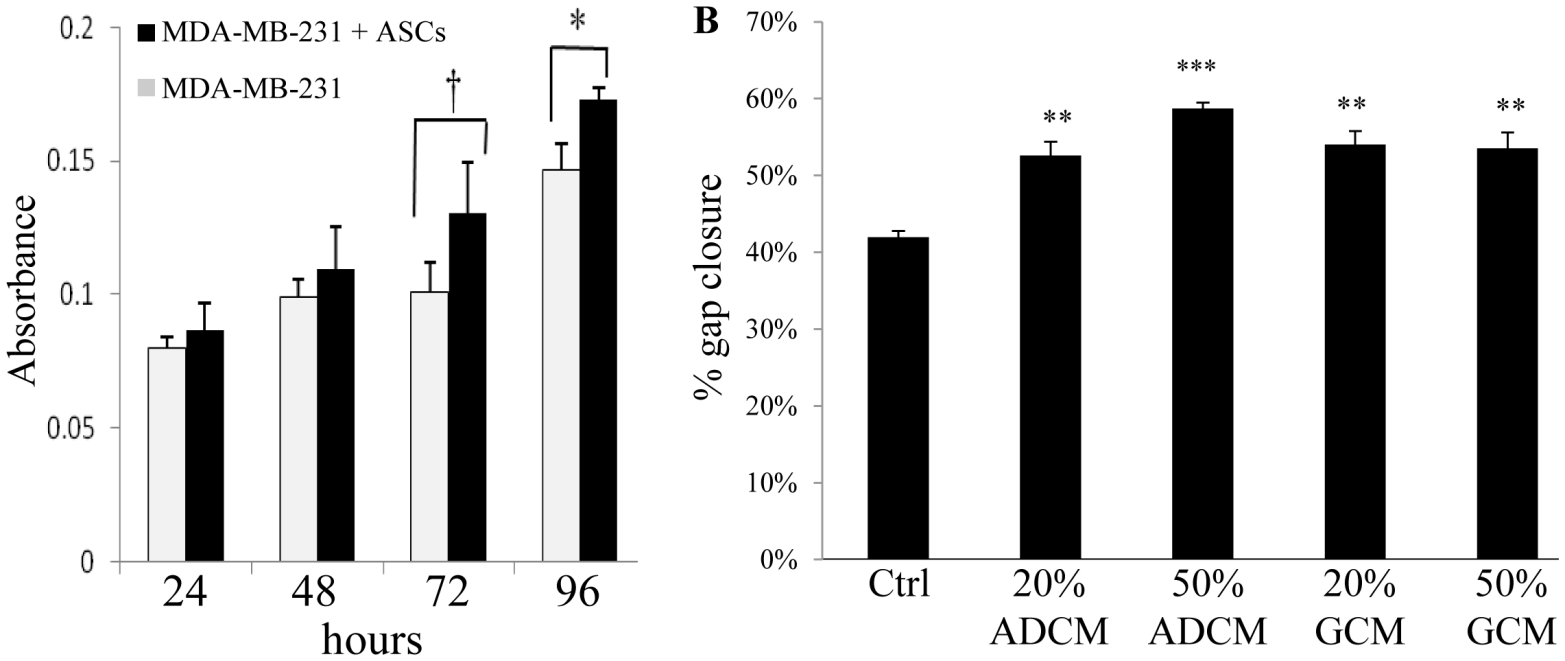
ASC effect on migration of MDA-MB-231 cells. A. ASCs were cultured in the bottom well of a Boyden Chamber and MDA-MB-231 cells were cultured in the insert. Migration of MDA-MB-231 cells was assessed by using crystal violet staining of the insert membrane and quantification of color development. †P<0.02, *P<0.04. **B.** MDA-MB-231 cells were cultured 24 h followed by replacement with medium containing 0%, 20% or 50% growth conditioned media (GCM) or adipocyte-differentiated conditioned medium (ADCM) from ASCs. A horizontal scratch was made using a P200 pipette tip and bright field pictures were taken at 0 and 6 h (Figure S1) following the scratch wound. Graphical representation of % gap closure quantitated using ImageJ software (NIH, Bethesda, MD). **P<0.01, ***P<0.0001. Data are representative of experiments using three different ASC donors.

A Boyden chamber was used to assess the effect of ASCs on MDA-MB-231 cell growth in indirect co-culture. At 24, 48 and 72 hours ASCs did not affect MDA-MB-231 growth (Figure 1A). 20% and 50% conditioned medium from three ASCs donors did not affect MDA-MB-231 cell growth *in vitro* but resulted in modest growth stimulation of MCF-7 (ER+/PR+) and BT-474 (ER+/PR+/HER2+) breast cancer cell lines (data not shown). To assess the effect of ASCs on MDA-MB-231 growth in direct co-culture, MDA-MB-231/GFP cancer cells were co-cultured with or without ASCs for 4 days and fluorescence microscopy was used to count the number of MDA-MB-231/GFP cells in the culture. There was no difference in the number of MDA-MB-231/GFP cells during 4 days co-culture with or without ASCs (Fig. 1B). Interestingly, fluorescent microscopy revealed that direct co-culture of MDA-MB-231 cells with ASCs resulted in a significant increase in the number of MDA-MB-231 cells that exhibited an elongated, spindle-like morphology reminiscent of migratory cells (Figure S1, white arrows). The ASC effect on migration of MDA-MB-231 cancer cells was assessed by indirect co-culture in a Boyden Chamber. After 72 h co-culture, ASCs stimulated migration of MDA-MB-231 cancer cells (Figure 2A). To assess whether the stimulation of MDA-MB-231 migration was the result of paracrine factors, CM from ASCs cultured in ASC growth medium, or CM from ASCs undergoing adipocyte differentiation was added to cultured MDA-MB-231 cells in the wound healing (scratch) assay. 20% and 50% CMs from proliferating ASCs or ASCs undergoing adipocyte differentiation stimulated migration of MDA-MB-231 cells (Figure 2B and Figure S2). As a control for the migration/invasion experiments, the effect of BJ5TA fibroblasts and THP-1 monocytes on the migration/invasion of MDA-MB-231 cells was assessed. BJ5TA fibroblasts or THP-1 monocytes did not alter MDA-MB-231 migration or invasion (data not shown). These data demonstrated that ASCs did not alter MDA-MB-231 breast cancer cell growth *in vitro* during direct or indirect co-culture. However, ASCs stimulated MDA-MB-231 cell migration and the effect was due, in part, to release of paracrine factors by the ASCs.

### ASC effect on primary MDA-MB-231 tumor growth was donor dependent

**Figure 3:**
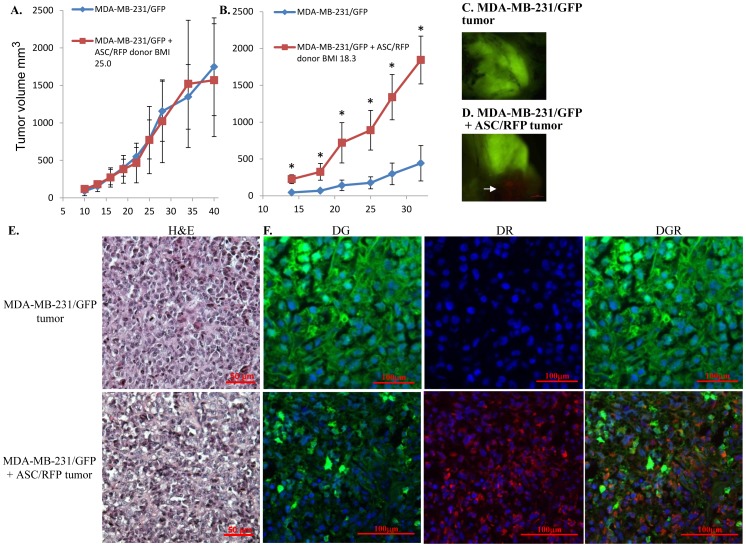
ASC effect on primary MDA-MB-231 xenografts. 3×10^6^ human MDA-MB-231/GFP breast cancer cells were bilaterally injected subcutaneously into the mammary fat pads of 5 female NUDE mice (n = 10 tumors/group) with or without 3×10^6^ human ASC/RFP cells from donor with BMI 25.0 (A) or donor with BMI 18.3 (B).Tumor volume was monitored for 40 days by caliper measurement. Tumors were removed at day 40 and fluorescence of the intact, fresh tumors from the MDA-MB-231/GFP alone group (**C**) or MDA-MB-231/GFP+ASC/RFP group (**D**) were visualized for GFP and RFP within 10 minutes of removal using a dissecting fluorescent microscope. The white arrow indicates a region of RFP fluorescence only in the MDA-MB-231/GFP+ASC/RFP group tumors. **E.** 5 µM paraffin embedded section of MDA-MB-231/GFP and MDA-MB-231/GFP+ASC/RFP tumors were prepared for Hematoxylin and Eosin (H&E) staining. **F.** 10 µM frozen sections of tumors were stained with DAPI (blue) and prepared for fluorescence microscopy for GFP and RFP. DAPI+GFP (DG); DAPI+RFP (DR); DAPI+GFP+RFP (DGR).

MDA-MB-231/GFP cells were co-injected with or without ASC/RFP cells (1∶1 ratio) isolated from two different donors with different body mass index (donor 1, female age 44, BMI 25.0, overweight; donor 2, female age 27, BMI 18.3, underweight) into the mammary fat pad of NUDE mice to determine the effect of ASCs on tumor growth and metastasis *in vivo*. Expression of GFP and RFP in MDA-MB-231/GFP cells and ASC/RFP cells, respectively, permitted monitoring of each cell type in the tumor and metastatic organs by fluorescent microscopy of whole organs and tissues sections. The MDA-MB-231 tumor xenograft is a very well characterized tumor model that spontaneously metastasizes from the primary tumor in the mammary fat pad of Nude mice [1], [53]. Injection of MDA-MB-231/GFP cells alone formed palpable tumors (Figure 3A). Injection of ASC/RFP cells alone did not form palpable masses in the mammary fat pad by 40 days (data not shown). Co-injection of BMI 25.0 ASC/RFP cells with MDA-MB-231/GFP cells did not alter the primary tumor volume or growth pattern up to 40 days post-injection with tumor sizes that were similar to MDA-MB-231/GFP alone tumors (Figure 3A; Figure S3A). However, coinjection of ASC/RFP cells from the BMI 18.3 donor with MDA-MB-231/GFP markedly stimulated growth of the tumors (Figure 3B; Figure S3B). The excised, fresh whole tumors from both groups exhibited a similar gross morphology with evidence of GFP expression throughout the tumors (Figure 3C, D). RFP expression was not readily apparent at the gross level due to masking by the GFP signal, although some regions of RFP were evident in regions of the tumor where the GFP expression was reduced (Figure 3D, arrow). No RFP expression was detected in whole tumors from the MDA-MB-231/GFP alone group.

### ASCs were integrated within the MDA-MB-231/GFP tumors

Tumor morphology was assessed by H&E staining, and fluorescence microscopy was used to distinguish MDA-MB-231/GFP cells from ASC/RFP cells. H&E staining revealed a similar tumor morphology for the MDA-MB-231/GFP group and the MDA-MB-231/GFP+ASC/RFP group (Figure 3E), that was also similar to the morphology of MDA-MB-231 tumors from our previous studies [1], [53]. The MDA-MB-231/GFP tumors exhibited GFP expression that overlapped with the majority of DAPI positively stained nuclei (Figure 3F). No RFP expression was detected in the tumors from the MDA-MB-231/GFP alone group. In the MDA-MB-231/GFP+ASC/RFP tumors, GFP expression and distinct RFP expression was detected in the same sections demonstrating evidence of viable RFP-expressing ASCs integrated throughout the tumor with an RFP signal that did not directly overlap with the GFP expressing cells at 40 days post injection (Figure 3F). There appeared to be less green fluorescent staining in tumor sections from the co-injection group compared to the MDA-MB-231 alone tumors although the reason for this is unknown. It was not possible to accurately quantitate the number of cancer cells or ASCs in the tumor sections. The fluorescent tissue sections were 10 µM thick and contained two or more cell layers with overlapping red and green fluorescent signals and individual cells that exhibited variable expression of GFP or RFP. In addition, there were focal regions of relatively more GFP or RFP staining in the tumor.

### ASCs promoted metastasis of MDA-MB-231 tumors to lung, liver and spleen

**Figure 4:**
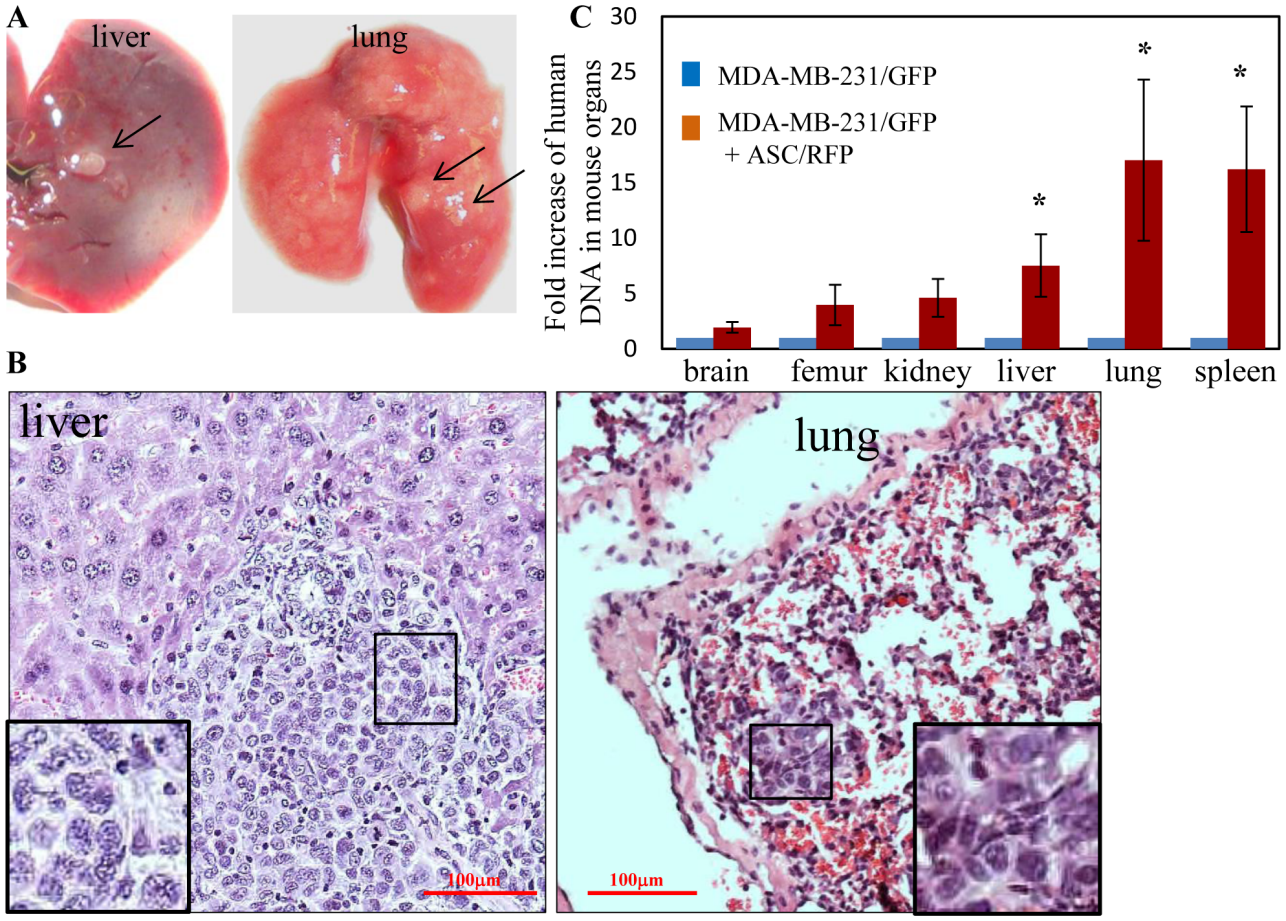
Metastasis of MDA-MB-231/GFP and MDA-MB-231/GFP+ASC/RFP tumors. 40 days after subcutaneous injection of either human MDA-MB-231/GFP cells, ASC/RFP cells or MDA-MB-231/GFP+ASC/RFP cells, mouse organs were collected. **A.** Visual macrometastatic lesions were observed in the liver, lungs only in mice co-injected with MDA-MB-231/GFP and ASC/RFP (arrows). **B.** H&E sections of the liver and lungs of mice bearing MDA-MB-231/GFP+ASC/RFP tumors showing metastatic MDA-MB-231 cancer cells (insets). **C.** To quantitate micrometastases, DNA was prepared from mouse organs from two separate experiments (n = 10 mice/group) for detection of human chromosome 17 by real time RT-PCR. A significant increase in micrometastasis for MDA-MB-231/GFP+ASC/RFP tumors was detected in liver, lung and spleen. * P<0.05.

MDA-MB-231 xenograft tumors in the mammary fat pad of Nude mice exhibit spontaneous micrometastases to select mouse organs that were dependent upon the primary tumor burden, and the duration of the experiment [1]. In the timeframe for development of large primary MDA-MB-231 tumors (30–40 days), no visible macrometastatic lesions were evident in mouse organs although micrometastases were detected by quantitation of the amount of human DNA in mouse organs by quantitative real-time RT-PCR directed towards an α-satellite sequence specific for human chromosome 17 [1], [53]. At the termination of experiments described in Figure 3, 6/10 animals in the MDA-MB-231/GFP+BMI 25.0-ASC/RFP group (from two separate experiments) showed evidence of visual macrometastases in the liver and lung (Figure 4A) as well as enlargement of the spleen (not shown) that was not evident in the MDA-MB-231/GFP group or the ASC/RFP group. To control for any leakage of cells into the systemic circulation as a result of the injection technique, PCR analysis for human chromosome 17 DNA in the blood one day after the injections was evaluated. A chromosome 17 DNA signal was not detected for any of the injection groups (data not shown). Fresh, intact lung, liver and spleen revealed GFP fluorescence in the MDA-MB-231/GFP+ASC/RFP group, but not the other groups (Figure S4). H&E stained paraffin sections of the liver and lung confirmed multi-focal metastatic lesions in the MDA-MB-231/GFP+BMI 25.0-ASC/RFP group only (Figure 4B). To further measure and compare the degree of micrometastasis among groups, brain, bone marrow from femurs, kidney, liver, lung and spleen were removed and the amount of human DNA in these mouse tissues was measured by quantitative real-time RT-PCR for human chromosome 17. The incidence of micrometastasis to all mouse organs/tissues was 10/10 mice (100%) for both the MDA-MB-231/GFP group and the MDA-MB-231/GFP+ASC/RFP group, and 0/10 (0%) for the ASC/RFP alone group. The relative level of human chromosome 17 DNA in each organ/tissue was compared for the MDA-MB-231/GFP group and the MDA-MB-231/GFP+ASC/RFP group, with the value for the MDA-MB-231/GFP group set equal to 1.0. A statistically significant increase in human chromosome 17 DNA was detected in liver, lung and spleen for the MDA-MB-231/GFP+ASC/RFP group (Figure 4C).

**Figure 5:**
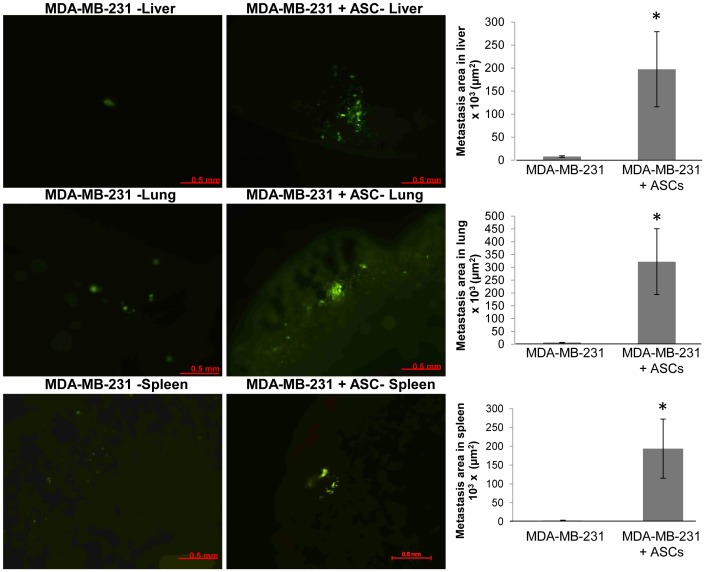
Detection of metastasis in whole organs by fluorescence quantitation. Metastases in fresh, whole organs were quantitated by detection of green fluorescence protein in mouse liver, lung and spleen. Image J software was used to quantitate the area of the fluorescent signal on the image as described in the Materials and Methods.

To further quantitate the degree of metastatic burden in these organs, the metastatic area was quantitated by measuring the total area of fluorescence in whole organs. The metastatic area in liver, lung and spleen was significantly greater in the MDA-MB-231+ASC group compared to the MDA-MB-231 alone group (Figure 5).

A control experiment in which an equal number of BJ5TA fibroblasts were co-injected with MDA-MB-231/GFP cells showed a modest effect on increasing primary tumor volume (Figure S5A, green line) but had no effect on metastasis to mouse organs as measured by the relative level of human chromosome 17 DNA in each organ/tissue (Figure S5B, green bars). To assess whether ASC donor impacted MDA-MB-231 metastasis, human chromosome 17 DNA was measured for the tumor xenograft experiment from Figure 3B using the BMI 25.0 ASCs. Tumors coinjected with BMI 25.0 ASCs resulted in increased metastasis to kidney, lung and spleen (Figure S5B, red bars). Similar to BMI 25.0 ASCs which increased MDA-MB-231 metastasis, coinjection with BM1 18.3 ASCs resulted in increased metastasis to lung, kidney and spleen (Figure S5B).

**Figure 6:**
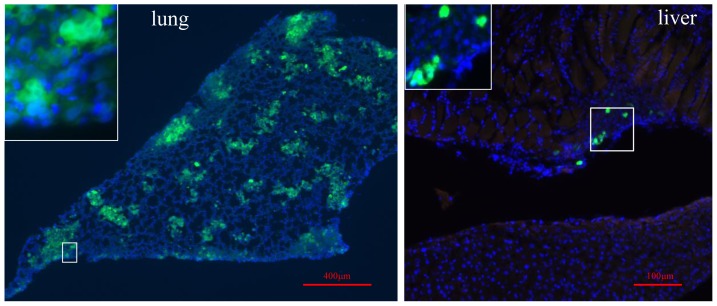
Metastatic lesions in lung and liver from the MDA-MB-231/GFP+ASC/RFP group tumors. 40 days after subcutaneous injection of MDA-MB-231/GFP+ASC/RFP cells, mouse organs were collected and 10 µM frozen sections were prepared for immunofluorescence of the lung and liver. A representative section of lung demonstrating multifocal metastatic lesions expressing GFP. A representative section of the liver demonstrated a small region expressing GFP. RFP was not detected above background level in any frozen tissue sections.

The remaining experiments were performed using the tumors derived from coinjection of BMI 25.0 ASCs with MDA-MB-231 cells. Metastases to lung and liver were confirmed by fluorescence microscopy of frozen sections. In the MDA-MB-231/GFP+ASC/RFP group, extensive GFP fluorescence was evident in the lungs demonstrating multifocal metastatic lesions (Figure 6). GFP focal lesions were detected in the livers of these animals but to a lesser extent that was detected in lung (Figure 6). No GFP fluorescence above background was detected in frozen sections of the spleen or in any other mouse tissues examined for this group. For the MDA-MB-231/GFP alone group, small isolated GFP positive lesions consisting of few cells were detected in the lungs (Figure S6) but not in any other mouse tissues. RFP fluorescence above background level was not detected in tissue sections from any mouse organ for any group. The absence of ASC/RFP fluorescence in the mouse organs, and the negative signal for the more sensitive human chromosome 17 DNA content measurement indicated that ASC/RFP cells had not migrated from the primary tumor site to the mouse organs.

### ASC induced changes in EMT, matrix metalloproteinases and angiogenesis in the primary tumors

**Figure 7:**
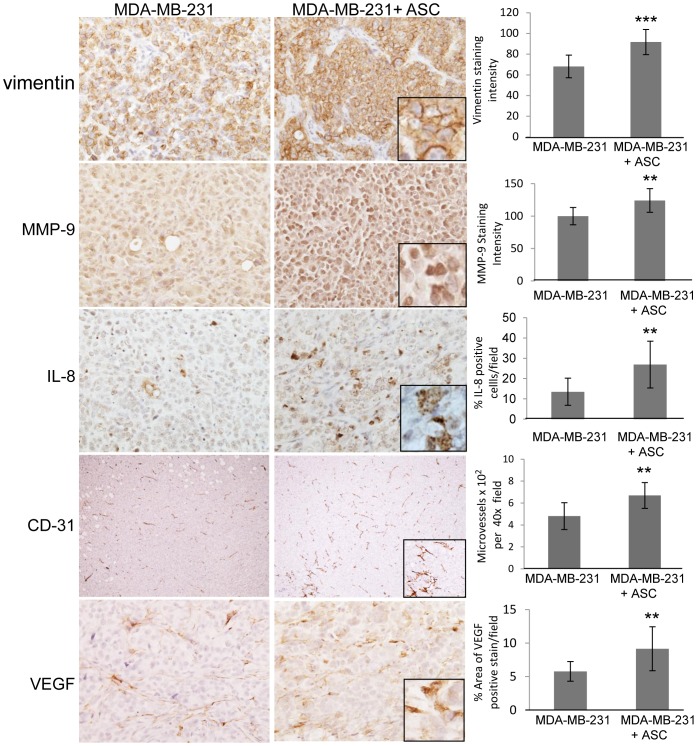
ASC effect on tumor markers. IHC was conducted as described in Materials and Methods.Paraffin-embedded tumor sections from MDA-MB-231/GFP and the MDA-MB-231/GFP+ASC/RFP groups were stained for vimentin, MMP9, IL-8, CD-31, and VEGF. Bright-field photomicrographs were taken and representative images are presented. Quantitative representation of the staining is indicated.

There are several possible mechanisms by which ASCs might increase the metastasis of MDA-MB-231 tumor cells, most notably induction of EMT in the tumor cells, increase in matrix metalloproteinases (MMP's), elevated angiogenesis in the tumors, and altered levels of paracrine factors. Tumors were compared for expression of EMT markers (vimentin, e-cadherin, beta catenin), MMP2/9, microvessel density, and paracrine factors (IL-8, VEGF). Tumors formed by coinjection of MDA-MB-231 with ASCs exhibited increased expression of vimentin, MMP9, IL-8, VEGF, and microvessel density (CD-31) (Figure 7).

## Discussion

The supplementation of fat grafts with ASCs to repair defects after breast cancer surgery has gained attention in recent years. ASC supplementation is proposed to increase the viability of the grafts and efficacy of the procedure. Recently, several laboratory studies demonstrated that ASCs stimulated breast cancer cell growth and migration *in vitro*, and co-injection of ASCs with breast cancer cells stimulated growth of xenograft tumors in mice that was accompanied by changes in behavior of the cancer cells and modification of the tumor stroma. The changes induced by ASCs were consistent with the cancer cells acquiring a more invasive, metastatic phenotype. These studies indicated the potential of ASCs to stimulate metastasis of breast tumors. The present study examined the impact of human ASCs on human MDA-MB-231 triple negative breast cancer cells, a model of early micrometastasis from the primary tumor. ASCs stimulated migration of MDA-MB-231 cells and markedly increased metastasis of MDA-MB-231 cells to mouse organs. Pathological evaluation of the primary tumors and the metastatic organs revealed that ASCs were well integrated in the primary tumor but were not present at the metastatic sites. The co-injection of ASCs with MDA-MB-231 cells resulted in tumors that exhibited some markers of EMT, angiogenesis and MMP expression, all consistent with a more invasive phenotype.

ASCs did not stimulate growth of MDA-MB-231 cancer cells *in vitro* using three separate ASC donors and in two different assays, although ASCs did modestly stimulate growth of MCF-7 and BT-474 breast cancer cell lines *in vitro*. Previous reports showed that ASCs or MSCs may stimulate growth or have no effect on MDA-MB-231 cell lines *in vitro* dependent upon the assays employed [29]–[31], [31]–[33], [33]–[35], [43], [44]. It is likely that the effects of ASCs on MDA-MB-231 cell growth are related to plating density, medium used, serum starvation status, ASC/MSC donor, and ASC passage number (ASCs used in the present study were at passage 1). In contrast to the *in vitro* results, there was a significant donor effect on the ability of ASCs to stimulate primary MDA-MB-231 tumor growth. ASCs from a donor with BMI 18.3 stimulated primary tumor growth whereas ASCs from a donor with BMI 25.0 did not stimulate tumor growth. Of note, neither of these ASC lines stimulated growth of MDA-MB-231 cells *in vitro* (Fig. 1). Future studies using multiple donors with different BMI will determine whether donor BMI impacts the effect of ASCs on tumor growth. There is conflicting evidence in the literature on the effect of ASCs or MSCs on primary MDA-MB-231 xenograft tumor growth. The present study was designed to form large, primary tumors in the mammary fat pad of NUDE mice that would yield metastases within 40 days [1]. Consequently, 3×10^6^ MDA-MB-231 and ASCs were injected into the mammary fat pad. In two studies that showed that ASCs stimulated MDA-MB-231 tumor xenograft growth, substantially fewer MDA-MB-231 cells and ASCs were injected into the mammary fat pad (10^3^–10^4^), the studies used SCID mice, and one study used the cleared mammary fat pad [31], [37]. Using bone marrow derived MSCs, Karnoub et al. co-injected a comparable number of MDA-MB-231 cells with MSCs (2×10^6^) as used in the present study into orthotopic sites and found no effect on MDA-MB-231 primary tumor growth [46]. Growth stimulation of the MDA-MB-231 xenografts by ASCs is likely dependent upon the initial tumor burden in the experiment. However the present study also demonstrated that ASC donor can have a significant effect on primary tumor growth.

Recently Strong et al. [58] demonstrated that abdominal ASCs derived from obese patients (BMI>30) enhanced MCF-7 ER-positive breast cancer cell proliferation *in vitro* and tumor xenograft growth in vivo. This study focused on ER positive breast cancer cells and it is unknown how ASC depot site and BMI of donors would impact ER negative tumors such as MDA-MB-231. However, the Strong et al. study also measured ASCs effect on MDA-MB-231 cell proliferation in vitro and found that pooled donors of ASCs (6/group) increased MDA-MB-231 cell growth approximately two fold after 7 days co-culture regardless of ASC depot site or donor BMI. Notably, the present study using three ASC donors (none derived from obese patients) co-cultured separately with MDA-MB-231 cells did not stimulate MDA-MB-231 growth after 3 days co-culture. The differences in the outcomes of two studies on MDA-MB-231 growth *in vitro* are likely due to differences in the assays (the Strong et al. study used longer co-culture time of 7 days versus 3 days, markedly lower seeding density of 200 cells/cm^2^ versus 2500/cm^2^, and pooled ASCs donors versus assessing individual ASC donors), and differences in the sources and passage number of ASCs donors and MDA-MB-231-GFP cells.

MDA-MB-231 tumor cells exhibit a migratory phenotype *in vitro* [1] although it has not been shown whether ASC/MSCs can enhance MDA-MB-231 migration *in vitro*. Surprisingly, incubation of ASCs with MDA-MB-231 cells in a Boyden chamber, or addition of ASC CM to MDA-MB-231 cells in culture, both further stimulated MDA-MB-231 migration. Of note was that the CM from both proliferating ASCs as well as ASCs undergoing adipogenic differentiation was able to stimulate migration of MDA-MB-231 cells. These results demonstrated that ASC stimulation of MDA-MB-231 migration was due to paracrine factors and these factors were present whether ASCs were undifferentiated or differentiated towards mature adipocytes. These data would suggest that the ASC adipocyte differentiation that may occur during applied use of ASCs might result in cells that are still competent to promote migration/metastasis of cancer cells. However, since ASCs that were differentiated *in vitro* also stimulated migration of MDA-MB-231 cells, we cannot conclude that properties unique to ASCs contributed to the increased migration. Furthermore, it should be noted that the conditioned medium was stored at 4C before use in these *in vitro* experiments and it is possible that key growth promoting factors might have been degraded and/or inactivated during storage. The increased migration was accompanied by a marked increase in the spindle-shape morphology of the MDA-MB-231 cells when in direct co-culture with ASCs. ASCs were integrated with the MDA-MB-231 cells *in vitro*, oftentimes surrounding clusters of MDA-MB-231 cells (Fig. S1). These data are consistent with recent reports that ASCs have a profound impact on the morphology of cancer cells, in some cases inducing an EMT in the cancer cells that enhanced cancer cell migration and invasion capacity [34], [35], [37].

It is clear from the tumor immunofluorescence sections that viable ASCs survived in the primary tumor at the time of sacrifice (40 days) and the ASCs were well integrated with the cancer cells in the tumor. The ASCs and MDA-MB-231 cells were mostly uniformly distributed in the tumor (Figure 4). There were sporadic areas in the tumors where ASC/RFP cells were detected separate from the MDA-MB-231/GFP cells but with no consistent pattern nor without any obvious morphology to indicate distinct structures or morphologies formed by the ASCs. The long term viability of the ASCs in the tumor is significant since co-injection with equal number of ASCs did not alter the primary tumor volume. This would suggest that a significant portion of the tumor volume was comprised of expanded ASCs, stroma from the ASCs, and/or recruited mouse cells/tissues. Recent studies have demonstrated that ASCs have a profound impact on the tumor stroma and that co-injected ASCs adopted a phenotype of cancer associated fibroblasts (CAFs) that could expand as occurs in the desmoplastic stromal reaction in breast cancer [59].

There appeared to be less green fluorescent staining in tumor sections from the co-injection group compared to the MDA-MB-231 alone tumors suggesting that there were fewer malignant cells in the co-injection group, although the reason for this is unknown. It was not possible to accurately quantitate the number of cancer cells or ASCs in the tumor sections. The fluorescent tissue sections were 10 µM thick and contained two or more cell layers with overlapping red and green fluorescent signals and individual cells that exhibited variable expression of GFP or RFP. In addition, there were focal regions of relatively more GFP or RFP staining in the tumor.

The significant increase in metastasis in the co-injection group for the BMI 25.0 ASCs occurred without any increase in primary tumor volume indicating that the elevated metastasis could not be attributed to an increased primary tumor burden in the animal. Visual metastases to the lungs and livers were only observed in the groups co-injected with MDA-MB-231/GFP cells and ASCs. Given the relatively brief duration of the experiment (40 days), the magnitude of the increased visible metastases is remarkable since no visual metastases were observed in any organs in the MDA-MB-231/GFP alone group. These data, along with the absence of an ASC effect on MDA-MB-231 growth *in vitro* suggest that a major effect of ASCs on MDA-MB-231 tumors is to promote the metastatic phenotype of the tumor cell. It is noted that mouse organs were rinsed thoroughly to remove blood prior to DNA isolation and quantitation of human chromosome 17 microsatellite regions. However one caveat is that any circulating tumor cells in the vasculature of the organs could contribute to the DNA quantitation as we have previously noted [1]. It is expected that given the high degree of metastatic involvement in many of the tissues, the contribution of any circulating tumor cells would be negligible.

The pattern of tumor cell dissemination to first pass organs (lung, liver, kidney and spleen), and the absence of ASC/RFP fluorescence in any mouse tissues suggested that the mechanism for ASC stimulation of metastasis was through enhancement of the early stages of the metastatic process in the primary tumor. The particularly high tumor burden in the lung (Figure 6) suggested that ASCs facilitated the escape of MDA-MB-231 tumor cells to the vasculature and lodgment in the lung, but without any accompanying ASCs. The increase in liver, kidney and spleen metastases without any corresponding ASCs in these tissues was also consistent with the absence of ASCs in the lung. The experiments were terminated at 40 days when the tumor burden became too large for the animal to survive. It is possible that given more time, additional organs would have exhibited an increased metastasis in the co-injection group.

There are several possible mechanisms by which ASCs might increase the metastasis of MDA-MB-231 tumor cells, most notably induction of EMT in the tumor cells, increase in MMP's, elevated angiogenesis in the tumors, and an increase in pro-metastatic paracrine factors. A number of studies have described secretion of paracrine factors by ASCs and related MSCs that stimulate cancer cell growth, migration/invasion and metastasis. ASCs produced SDF-1 [31], PDGF-D [34], IL-6 [60] and IL-8 [33] that contributed to growth and invasion/metastasis of breast cancer cells *in vitro* and *in vivo*. MSCs produced CCL5 that stimulated growth and metastasis of MDA-MB-231 tumor xenografts [34], and ASCs produced CCL5 that stimulated migration and invasion of breast cancer cells *in vitro* [32]. Conditioned medium from ASCs containing PDGF-D induced the mesenchymal markers fibronectin, alpha smooth muscle actin, and vimentin in breast cancer cells *in vitro* [34]. Conditioned medium from MSCs resulted in increased expression of mesenchymal markers N-cadherin, vimentin, twist and snail and downregulation in E-cadherin [41]. ASCs/MSCs may also stimulate angiogenesis and matrix degradation that can potentiate cancer cell metastasis. MSCs stimulated MMP-11 and VEGF in breast cancer cells [41]. Lipoaspirates of white adipose tissue were found to contain CD34+ progentiors that contributed to tumor vascularization [61]. In the present study we assessed several markers of EMT, matrix degradation, paracrine factors and angiogenesis and found that tumors formed by co-injection with ASCs exhibited markers consistent with these phenotypes.

In summary, the present study demonstrated that human ASCs markedly increased migration and metastasis of human MDA-MB-231 cancer cells in a xenograft model that was likely due to facilitation of the early steps of the metastatic process. These data, along with several recent reports demonstrating that ASCs induced EMT in tumor cells and alterations in tumor stroma consistent with metastatic progression [34], [35], [37], suggest that caution is warranted in applied use of ASCs in close proximity to breast cancer cells that have a greater propensity to metastasize.

## Supporting Information

Figure S1 **Co-culture of MDA-MB-231/GFP cells with ASCs.** MDA-MB-231/GFP cells (2.5×10^4^ cells/well) or MDA-MB-231/GFP+ASCs (at a 1∶1 ratio) were cultured in 6 well plates for 4 days and bright field and fluorescent microscopy photographs were taken on day 4. White arrows indicate an increased number of MDA-MB-231/GFP cells that exhibited elongated, spindle-like morphology when co-cultured with ASCs (red arrows). White box inset indicates MDA-MB-231/GFP cells. Red box inset indicates ASCs.
(PDF)

Figure S2 **Light micrographs for wound healing assay described in** **Figure 2B** **.** Growth conditioned media (GCM) and adipogenic-differentiated conditioned media (ADCM) from ASCs increased migration of MDA-MB-231 breast cancer cells. MDA-MB-231 cells were cultured for 24 h followed by replacement with medium containing 0%, 20% or 50% GCM or ADCM and a horizontal scratch using a P200 pipette tip. Pictures were taken 0 and 6 hrs. tumors following the scratch wound.
(PDF)

Figure S3 **Light micrographs of MDA-MB-231/GFP and the MDA-MB-231/GFP+ASC/RFP tumors excised at the termination of the experiments using ASC/RFP donor BMI 25.0 (A) or ASC/RFP donor BMI 18.3 (B).**
(PDF)

Figure S4 **Whole organ fluorescence from animals injected with MDA-MB-231/GFP+ASC/RFP cells.** Mouse organs were removed at day 40 and fluorescence of the fresh, intact mouse lung, liver and spleen were visualized for GFP and RFP within 10 minutes of removal using a dissecting fluorescent microscope. Fresh, intact organs from non-injected animals did not exhibit fluorescence (not shown).
(PDF)

Figure S5 **Effect of BJ5TA fibroblasts and BMI 18.3 ASCs on primary MDA-MB-231 tumor volume and metastasis.** 3×10^6^ human MDA-MB-231/GFP breast cancer cells were bilaterally injected subcutaneously into the mammary fat pads of 5 female NUDE mice (n = 10 tumors/group) with or without 3×10^6^ human BJ5TA fibroblasts or 3×10^6^ human BMI 18.3 ASCs. Tumor volume was monitored by caliper measurement. (**A**) Tumor volume of MDA-MB-231/GFP tumors and MDA-MB-231/GFP+BJ5TA fibroblasts tumors. (**B**) To quantitate micrometastases, DNA was prepared from mouse organs (brain, femur, kidney, liver, lung, spleen) from the three groups (MDA-MB-231/GFP alone, MDA-MB-231/GFP+BJ5TA fibroblasts, and MDA-MB-231/GFP+BMI 18.3 ASCs) for detection of human chromosome 17 by real time RT-PCR. * p<0.05.
(PDF)

Figure S6 **MDA-MB-231/GFP metastatic cells detected in lung from MDA-MB-231/GFP group tumors.** MDA-MB-231/GFP tumors (without co-injected ASC/RFP cells) resulted in only isolated nests of tumor cells in the lung but not in other tissues. Shown is one micrometastatic lesion in the lung comprising 10–12 GFP positive cells. GFP (G); RFP (R); DAPI (D); DAPI+GFP+RFP (DGR).
(PDF)
