# Supplementary figures and images for: Human Adipose Tissue-Derived Stromal/Stem Cells Promote Migration and Early Metastasis of Triple Negative Breast Cancer Xenografts
Source: PLoS One. 2014 Feb 28;9(2):e89595. doi: 10.1371/journal.pone.0089595 (PMC3938488; doi:10.1371/journal.pone.0089595)

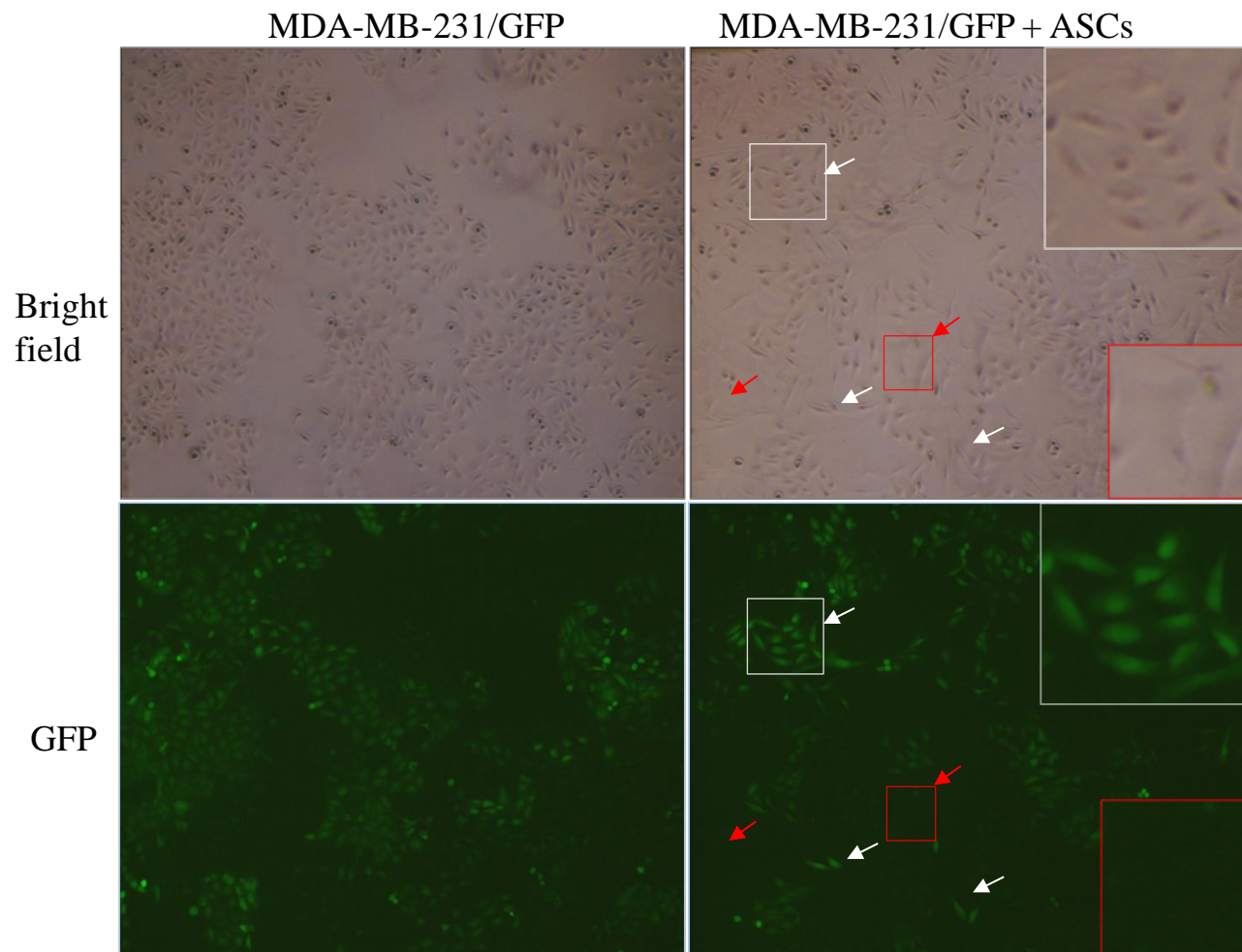

**Figure S1**

Supplement: Figure S1 — Co-culture of MDA-MB-231/GFP cells with ASCs. MDA-MB-231/GFP cells (2.5×10^4^ cells/well) or MDA-MB-231/GFP+ASCs (at a 1∶1 ratio) were cultured in 6 well plates for 4 days and bright field and fluorescent microscopy photographs were taken on day 4. White arrows indicate an increased number of MDA-MB-231/GFP cells that exhibited elongated, spindle-like morphology when co-cultured with ASCs (red arrows). White box inset indicates MDA-MB-231/GFP cells. Red box inset indicates ASCs. (PDF) [file pone.0089595.s001.pdf]

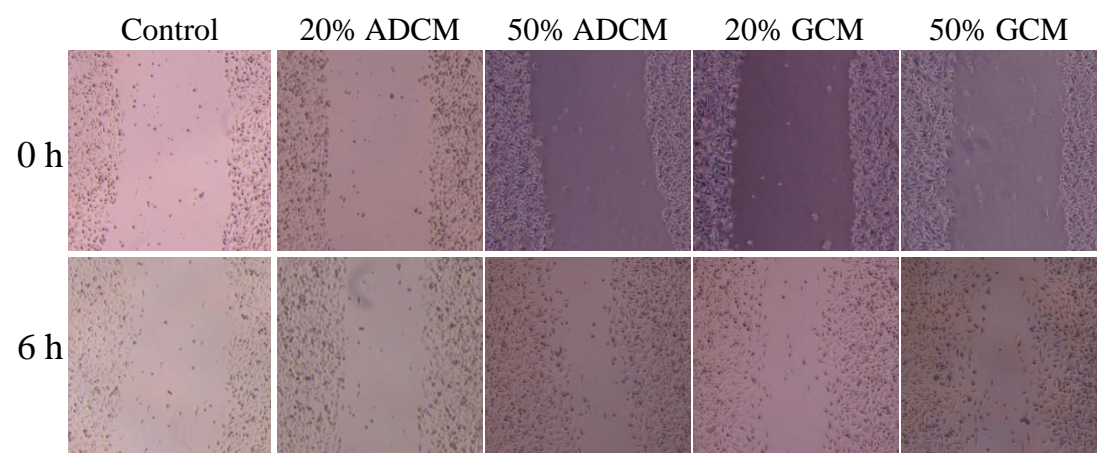

**Figure S2**

Supplement: Figure S2 — Light micrographs for wound healing assay described in Figure 2B . Growth conditioned media (GCM) and adipogenic-differentiated conditioned media (ADCM) from ASCs increased migration of MDA-MB-231 breast cancer cells. MDA-MB-231 cells were cultured for 24 h followed by replacement with medium containing 0%, 20% or 50% GCM or ADCM and a horizontal scratch using a P200 pipette tip. Pictures were taken 0 and 6 hrs. tumors following the scratch wound. (PDF) [file pone.0089595.s002.pdf]

## A. MDA-MB-231/GFP

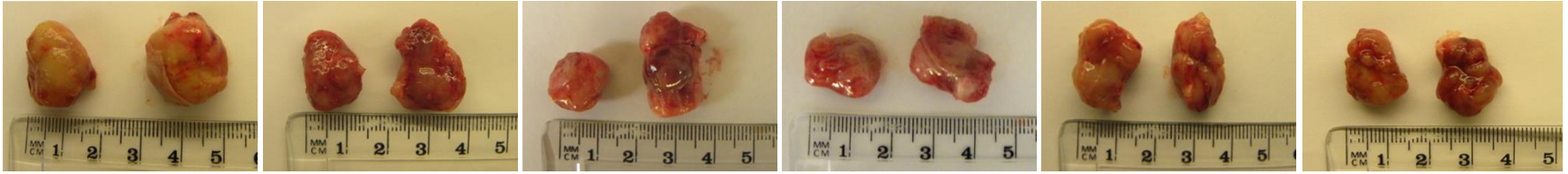

MDA-MB-231/GFP + ASC/RFP donor BMI 25.0

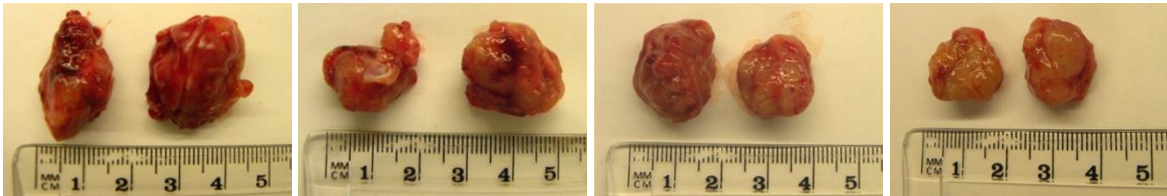

## B. MDA-MB-231/GFP

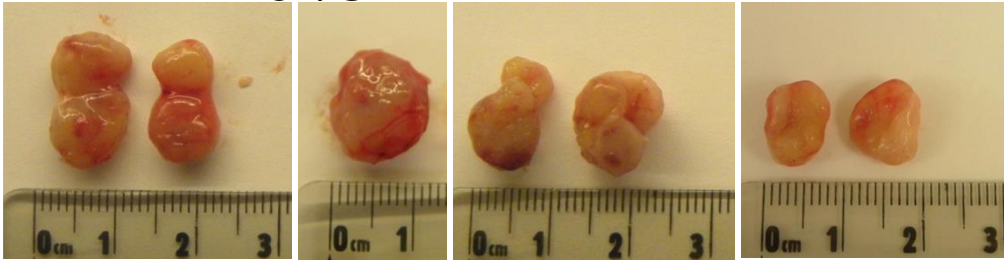

MDA-MB-231/GFP + ASC/RFP donor BMI 18.3

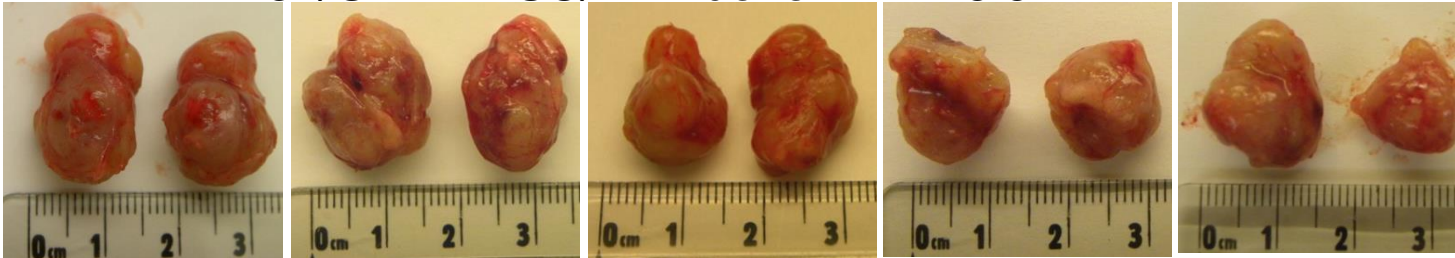

Figure S3

Supplement: Figure S3 — Light micrographs of MDA-MB-231/GFP and the MDA-MB-231/GFP+ASC/RFP tumors excised at the termination of the experiments using ASC/RFP donor BMI 25.0 (A) or ASC/RFP donor BMI 18.3 (B). (PDF) [file pone.0089595.s003.pdf]

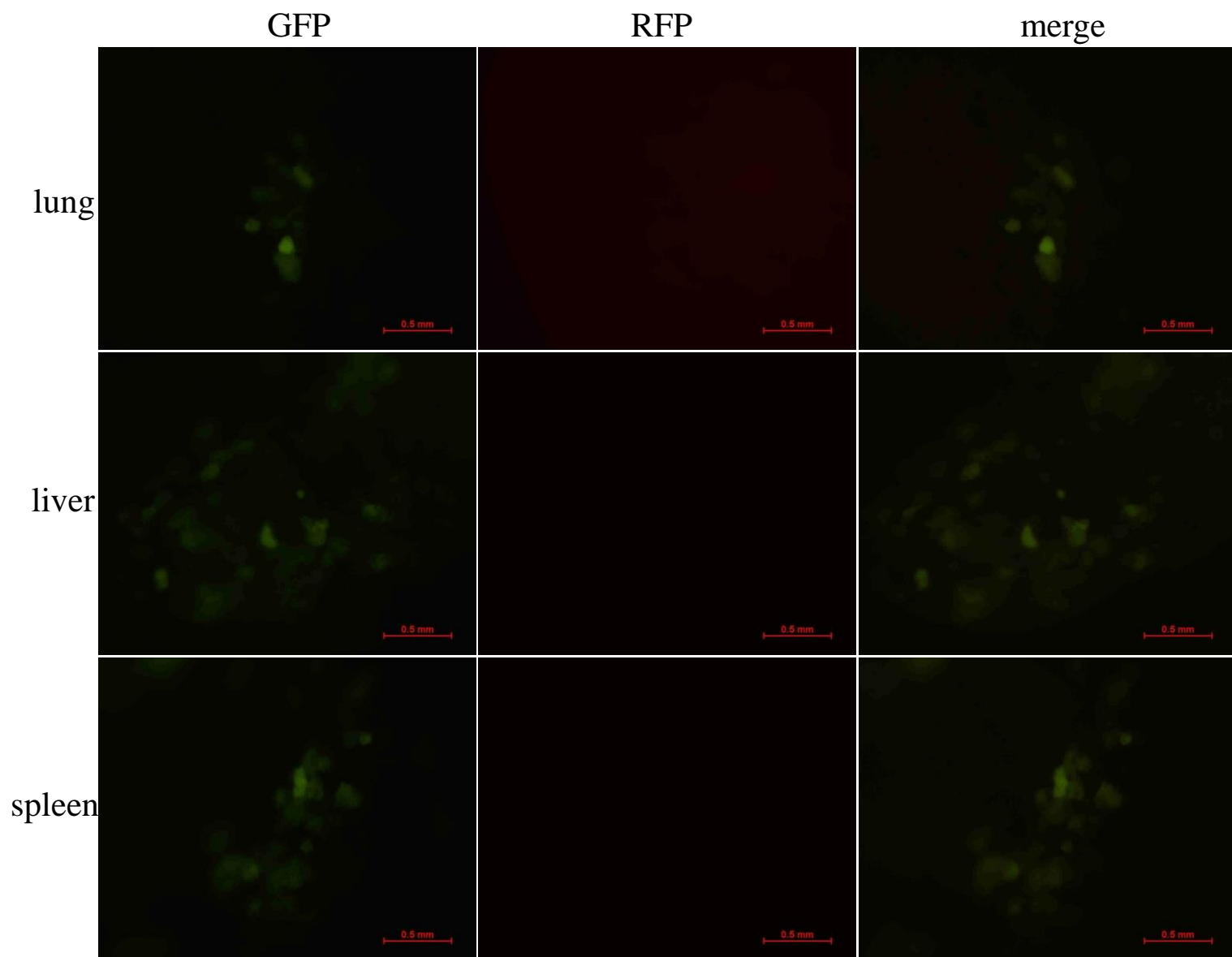

**Figure S4**

Supplement: Figure S4 — Whole organ fluorescence from animals injected with MDA-MB-231/GFP+ASC/RFP cells. Mouse organs were removed at day 40 and fluorescence of the fresh, intact mouse lung, liver and spleen were visualized for GFP and RFP within 10 minutes of removal using a dissecting fluorescent microscope. Fresh, intact organs from non-injected animals did not exhibit fluorescence (not shown). (PDF) [file pone.0089595.s004.pdf]

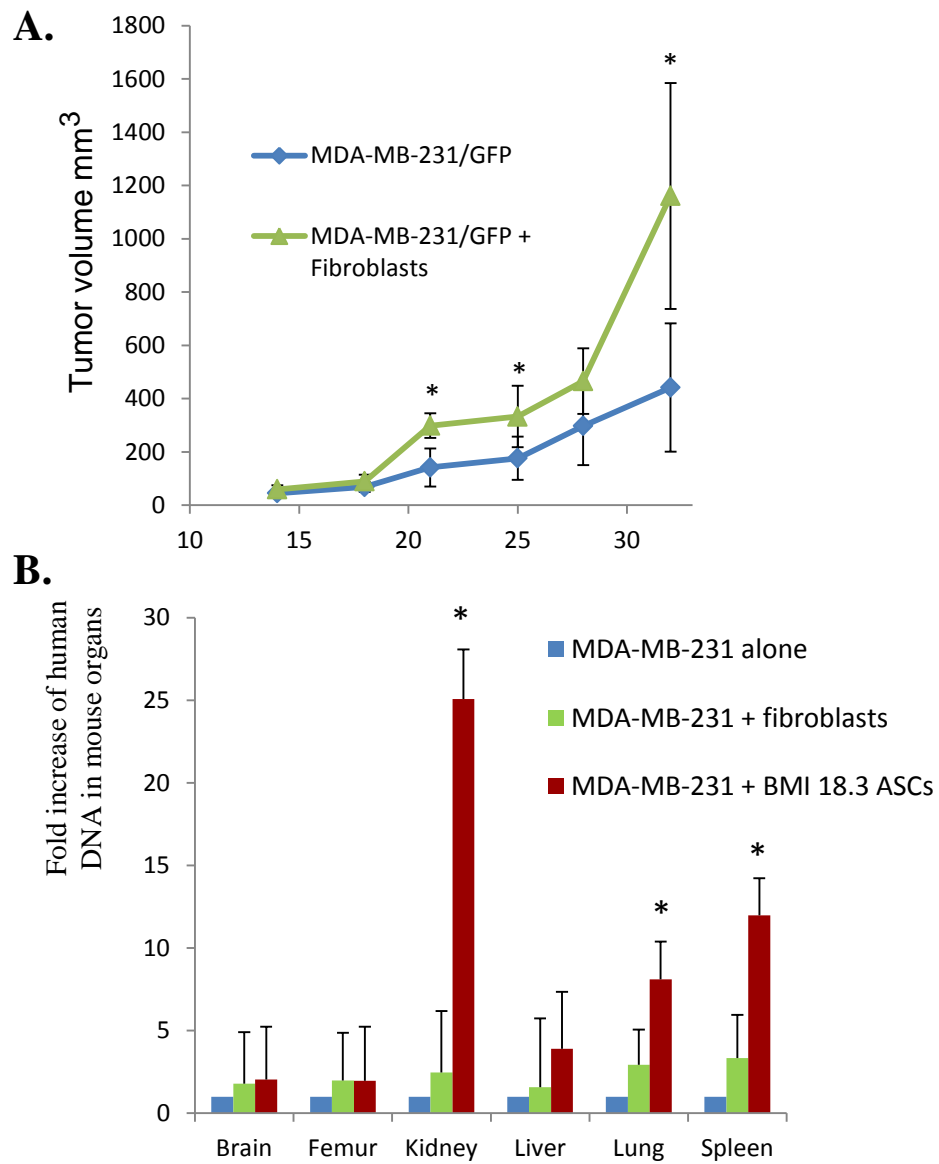

Figure S5

Supplement: Figure S5 — Effect of BJ5TA fibroblasts and BMI 18.3 ASCs on primary MDA-MB-231 tumor volume and metastasis. 3×10^6^ human MDA-MB-231/GFP breast cancer cells were bilaterally injected subcutaneously into the mammary fat pads of 5 female NUDE mice (n = 10 tumors/group) with or without 3×10^6^ human BJ5TA fibroblasts or 3×10^6^ human BMI 18.3 ASCs. Tumor volume was monitored by caliper measurement. (A) Tumor volume of MDA-MB-231/GFP tumors and MDA-MB-231/GFP+BJ5TA fibroblasts tumors. (B) To quantitate micrometastases, DNA was prepared from mouse organs (brain, femur, kidney, liver, lung, spleen) from the three groups (MDA-MB-231/GFP alone, MDA-MB-231/GFP+BJ5TA fibroblasts, and MDA-MB-231/GFP+BMI 18.3 ASCs) for detection of human chromosome 17 by real time RT-PCR. * p<0.05. (PDF) [file pone.0089595.s005.pdf]

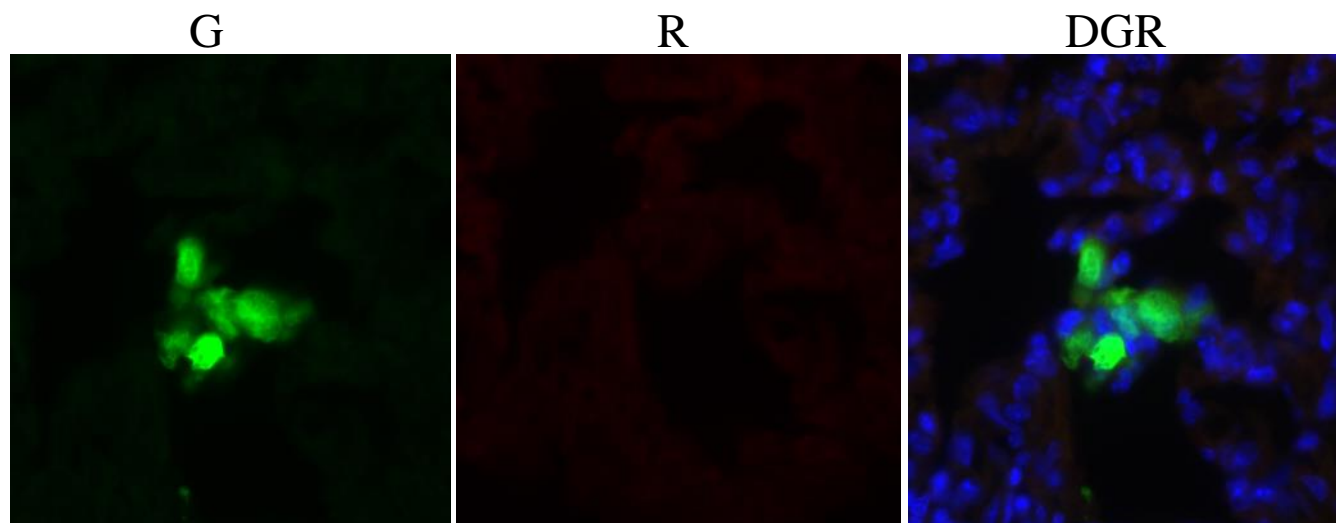

**Figure S6**

Supplement: Figure S6 — MDA-MB-231/GFP metastatic cells detected in lung from MDA-MB-231/GFP group tumors. MDA-MB-231/GFP tumors (without co-injected ASC/RFP cells) resulted in only isolated nests of tumor cells in the lung but not in other tissues. Shown is one micrometastatic lesion in the lung comprising 10–12 GFP positive cells. GFP (G); RFP (R); DAPI (D); DAPI+GFP+RFP (DGR). (PDF) [file pone.0089595.s006.pdf]
